# Early insights into the potential of the Oxford Nanopore MinION for the detection of antimicrobial resistance genes

**DOI:** 10.1093/jac/dkv206

**Published:** 2015-07-28

**Authors:** Kim Judge, Simon R. Harris, Sandra Reuter, Julian Parkhill, Sharon J. Peacock

**Affiliations:** 1 Department of Medicine, University of Cambridge, Cambridge, UK; 2 Wellcome Trust Sanger Institute, Hinxton, UK; 3 Public Health England, Clinical Microbiology and Public Health Laboratory, Cambridge, UK

## Abstract

**Objectives:**

Genome sequencing will be increasingly used in the clinical setting to tailor antimicrobial prescribing and inform infection control outbreaks. A recent technological innovation that could reduce the delay between pathogen sampling and data generation is single molecule sequencing. An example of this technology, which is undergoing evaluation through an early access programme, is the Oxford Nanopore MinION.

**Methods:**

We undertook a feasibility study on six clinically significant pathogens, comparing the MinION to the Illumina MiSeq and PacBio RSII platforms. Genomic DNA was prepared and sequenced using the MinION as instructed by the manufacturer, and Illumina MiSeq and PacBio sequencing was performed using established methods.

**Results:**

An evaluation of the accuracy of the MinION based on sequencing of an MRSA isolate showed that error rates were higher in the MinION reads, but provided an even coverage across the entire genome length. The MinION detected all of the expected carbapenemases and ESBL genes in five Gram-negative isolates and the *mecA* gene in an MRSA isolate.

**Conclusions:**

The MinION can detect the presence of acquired resistance genes, but improvements in accuracy are needed so that antimicrobial resistance associated with mutations in chromosomal genes can be identified.

## Introduction

The emergence of mobile genetic elements encoding ESBL and carbapenemases in Gram-negative bacilli represents a major threat to human health.^1^ These elements have the potential to spread within and between bacterial species, and have been associated with outbreaks in vulnerable populations.^2^ Rapid molecular-based prediction of phenotypic drug susceptibility could facilitate targeted prescribing and forms one element of a multifaceted approach to antibiotic stewardship. Achieving this using WGS technology has the potential advantage that this reveals the entire gene repertoire, and if linked to automated interpretation tools would represent an improvement on other molecular methods such as PCR that target specific gene panels. A recent technological innovation that has the potential to generate rapid sequence data is single-molecule sequencing, and a device based on this approach (Oxford Nanopore MinION) is under evaluation by the scientific community through an early access programme. This device is about the size of a mobile phone, connects to a computer via a USB port, and streams data via the Internet to the base-calling software. The first publication of its performance using the version R6 flow cell reported high error rates, with only 10% of reads from a 36 h sequencing run of lambda phage mapping to the reference, and <1% of the data generated matching the reference.^3^ A second publication reported whole-genome shotgun sequencing of *Escherichia coli* using the R7 and R7.3 flow cells, in which the authors concluded that further work was required to determine appropriate algorithms for common analyses.^4^ A subsequent publication reported the use of the MinION to resolve the structure and chromosomal insertion site of a composite antibiotic resistance island in *Salmonella* Typhi Haplotype 58.^5^ Recently, the ability of the MinION to rapidly distinguish outbreak from non-outbreak strains of *Salmonella* Enteritidis was evaluated as part of a wider study into a *Salmonella* outbreak.^6^ Here, we report the results of a comparative study of clinically significant antibiotic-resistant pathogens, in which sequence data generated by the MinION were compared with those generated by the Illumina MiSeq and PacBio RSII platforms.

## Methods

### DNA extraction and sequencing

Bacterial genomic DNA was extracted using the QiaAMP DNA Mini kit (Qiagen, Venlo, Limburg, the Netherlands). MinION sample preparation was performed according to the manufacturer's instructions and sequenced on R7 flow cells. The sequencing script was run in MinKNOW V0.45.3.9, and Metrichor V0.17 was used for basecalling using the R7 1.2 and 1.3 2D workflows. Illumina sequencing was carried out as described previously. PacBio SMRT (single-molecule real-time) sequencing was used to generate long reads of MRSA SASCBU26. Library preparation (SMRTbell) was carried out following the manufacturer's protocol (Pacific Biosciences, Menlo Park, CA, USA). The SMRTbell library was bound with version P4 polymerase, and the subsequent complexes loaded on to V3 SMRTcells using MagBeads. The complexes were immediately sequenced using version C2 chemistry. All sequence data have been submitted to the European Nucleotide Archive (ENA) (http://www.ebi.ac.uk/ena/) (see Supplementary Methods for details, available as Supplementary data at *JAC* Online).

### Analysis

Basecalled MinION reads were converted from FAST5 to FASTQ and FASTA formats using an in-house script. Read mapping of the MRSA isolate was carried out using the BWA-MEM algorithm of BWA v0.7.10. For short read Illumina data, the default mapping parameters were applied. For MinION and PacBio data, the PacBio mapping option was used, with a minimum seed length variation of between 7 and 21 to optimize the mapping. The presence of resistance genes were identified in Gram-negative bacilli and MRSA using a clustered version of the ARG-ANNOT resistance gene database.^7^ This was searched against the MinION reads using glsearch v36.3.5e from the FASTA package to identify matches between the entire length of each gene in the database and local regions of the MinION reads. For MRSA SASCBU26, the same method was used to identify gene matches in PacBio reads. Illumina data for each isolate were assembled and gene matches were identified in the assembly using glsearch with an e-value cut-off of 1e-100 and a %ID cut-off of 70. Detailed methods are provided in the Supplementary Methods section.

## Results

First, we evaluated the accuracy of the MinION based on WGS of an MRSA isolate (SASCBU26). Combined data from two MinION R7 flow cells were compared with sequence data generated previously using the Illumina MiSeq and PacBio RSII platforms.^8,9^ Sequence data from all three platforms were mapped to a reference genome generated using Illumina and PacBio data. Alignment of MinION data proved difficult because of a high error rate, and parameters were chosen that were comparable to those used for mapping the PacBio data (see Supplementary Methods for details), which produced a relatively stringent alignment. Under these mapping criteria only 25% of the MinION reads aligned to the reference, although this provided an even mapping depth across the genome (Table 1). Error rates were substantially higher in the mapped MinION reads compared with the two other technologies, with short indels being much more common in the MinION data (Table 1). There was less difference in the rate of erroneous base calls (substitution errors) between the three platforms, and such errors may have been artificially raised due to misalignments caused by the high level of indels in the MinION data.

**Table 1.** Reads generated by MinION (top) and comparison with PacBio RSII and MiSeq (bottom) for DNA extracted from an MRSA isolate

	MinION read type		
	all reads	all 2D	all 1D
Total	74 748	14 436	60 312
Mean length	1857	2965	1592
Median length	2910	3233	2762
	Sequencing platform		
	RSII	MiSeq	MinION (all mapped reads)
Depth	130×	72×	12×
Standard deviation	40×	35×	4.9×
Percentage covered	100	100	99.97
Insertions	0.008	2 × 10^−6^	0.107
Deletions	0.007	1 × 10^−5^	0.115
Errors	0.008	0.002	0.03

**Figure 1.**
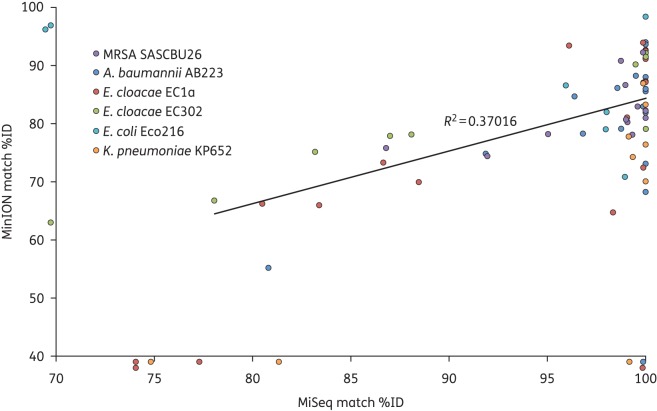
Scatter plot of %ID of gene cluster matches. Coloured points represent the %ID of gene cluster matches found in each isolate, as denoted in the inset key. %ID of matches identified in the Illumina assemblies is shown on the *x*-axis and the %ID of the consensus of matches identified in MinION reads is shown on the *y*-axis. Points plotted below the *x*-axis indicate no match in the MinION data, while those to the left of the *y*-axis indicate no match in the Illumina assembly.

Next, we evaluated the performance of the MinION against the MiSeq platform for the detection of acquired genes associated with clinically significant antimicrobial resistance using a panel of six isolates. Five were Gram-negative bacterial species that had been sequenced previously, and were known to possess phenotypic antibiotic resistance and a range of plasmid-mediated ESBLs and carbapenemases, as follows: *Acinetobacter baumannii* AB223 positive for an OXA-23 carbapenemase; *Enterobacter cloacae* EC1a positive for an IMP-1 carbapenemase; *Klebsiella pneumoniae* KP652 and *E. coli* Eco216 both positive for CTX-M-15 ESBL; and *E. cloacae* EC302, which was previously reported not to contain any ESBL or carbapenemase genes.^2^ The sixth isolate was MRSA SASCBU26, which is positive for the methicillin resistance gene (*mecA*) present on a chromosomally integrated SCC*mec* type IVc element.^8^ Miseq data were assembled as described in the Supplementary Methods section. It was not possible to assemble MinION reads due to the high error rate but, as the long reads generated by the MinION often span the length of a gene, the full gene sequence was available from read data without assembly. A consensus sequence was called for each gene as described in the Supplementary Methods section. Sequence data generated by the MiSeq and MinION were independently interrogated for the presence of genes encoding resistance using a publicly available database of bacterial antimicrobial resistance genes (ARG-ANNOT, Méditerranée Infection). A clustered version of this database was used, in which gene sequences in the database with 90% sequence identity or greater have been clustered together.^7^ In total, 75 gene cluster matches were identified for the six isolates, of which 64 (85%) matches were found in both the MinION and MiSeq results, and 11 were identified in data generated by one of the two technologies. Table S1 shows further detail of the matches found for each isolate in the MiSeq and MinION data. All of the expected carbapenemases and ESBL genes in the Gram-negative isolates and the *mecA* gene in the MRSA isolate were identified from MiSeq and MinION data. Comparison of the percentage identity (%ID) of the matches common to MiSeq and the MinION data for each isolate demonstrated a correlation between the two (Figure 1; *R*^2^ = 0.37).

Eight matches were only identified in the MiSeq data, five of which were low-identity matches (<85%). The MinION data yielded three matches not found in the MiSeq data, of which one was found in a single read with a very low identity (52%) to an AmpH β-lactamase cluster. Both the MiSeq and MinION data for this isolate identified matches to a second AmpH β-lactamase cluster, suggesting that this represented assignment of the read to the wrong cluster. The remaining two matches found in the MinION data, but not the corresponding MiSeq data, were high-identity matches (>87%) to TEM-1D and *aac3-IIa* in the data from the *E. coli* isolate Eco216. By checking the MiSeq data generated for the same sample in a previous study,^2^ we confirmed the presence of these genes. Both genes are commonly plasmid encoded, suggesting that the plasmid was lost during preparation of DNA for our new MiSeq sequencing run. Conversely, a plasmid-encoded β-lactamase gene (*bla*_MIR_) that had gone undetected in our previous study^2^ was identified in *E. cloacae* EC302 from both the MiSeq and MinION data. PacBio data were also available for SASCBU26, which allowed a comparison with MinION. This demonstrated an identical set of matches to those found in the MinION reads (Table S1). There was a correlation between the number of MinION reads matching a gene and the %ID of the match to the database (Figure S1).

## Discussion

The on-going development of a range of sequencing instruments provides an opportunity to match the needs of a specific application to performance qualities. Sequencing for clinical purposes must be both rapid and accurate if the results are to lead to information that changes the course of individual patient care or a public health intervention. The MinION can detect the presence of clinically important acquired resistance genes, but does not provide sufficient accuracy to detect resistance associated with point mutations in chromosomal genes. The inability to detect point mutations in relation to resistance is not only relevant for chromosomal genes as detection of sub-variants within a gene (cluster) can be very important, for example when distinguishing between TEM-1 and TEM52.^10^ Affordability is also an essential criterion for NHS laboratories and other publically funded institutions. The MiSeq Dx sequencing instrument, approved for clinical use by the FDA, retails at around $125 000.^11^ The MinION has low capital costs, with current sample preparation requiring minimal equipment. Oxford Nanopore have proposed a model for the future in which users would be charged based on ‘pay-as-you-go’, with 3 h of sequencing on the MinION MkI projected to cost around $270.^12^ One potential advantage of the MinION is its portability and ease of use, which could facilitate its uptake by diagnostic laboratories that anticipate low demand for microbial sequencing, but choose to develop in-house sequencing capabilities. Based on portability, the MinION could also find utility in more remote laboratories, particularly where pathogen sequencing could be used to investigate emerging pathogens and outbreaks of unknown cause.

A potential benefit of single-molecule sequencing is the speed at which sequence data could be generated, and the fact that data can be analysed in real time. In the event that high-quality data could be generated within the first few hours of a sequencing run, this could be combined with other methods such as rapid DNA extraction from bacterial colonies grown from the primary sample to minimize time from culture to sequence data.^13^ However, the major barrier to clinical implementation is the availability of automated interpretation tools—which affects all platforms equally. These early evaluations suggest that direct sequencing using a mobile device could find utility in diagnostic microbiology, given further improvements to speed, accuracy and yield.

## Funding

This study was funded by a grant from the Department of Health, the Wellcome Trust and the Health Innovation Challenge Fund (HICF-T5-342 and WT098600; to S. J. P.). S. R. H. and J. P. were supported by Wellcome Trust grant 098051. The MinION and reagents supplied by Oxford Nanopore were received as part of Oxford Nanopore's MinION access programme.

## Transparency declarations

J. P. and S. J. P. have received funding for travel and accommodation from Illumina Inc. All other authors: none to declare.

## Supplementary data

Supplementary Methods, Table S1 and Figure S1 are available as Supplementary data at *JAC* Online (http://jac.oxfordjournals.org/).

Supplementary Data
