# Supplementary material for: Early insights into the potential of the Oxford Nanopore MinION for the detection of antimicrobial resistance genes
Source: J Antimicrob Chemother. 2015 Jul 28;70(10):2775–8. doi: 10.1093/jac/dkv206 (PMC4566964; doi:10.1093/jac/dkv206)
Supplement: Supplementary Data [file supp_70_10_2775__index.html]

Early insights into the potential of the Oxford Nanopore MinION for the detection of antimicrobial resistance genes — Early insights into the potential of the Oxford Nanopore MinION for the detection of antimicrobial resistance genes — Early insights into the potential of the Oxford Nanopore MinION for the detection of antimicrobial resistance genes — Supplementary Data 

# Early insights into the potential of the Oxford Nanopore MinION for the detection of antimicrobial resistance genes

## Supplementary Data

Supplementary Data

- Supplementary Data - Docx file
- Supplementary Table 1 - xlsx file
