# Supplementary material for: Early insights into the potential of the Oxford Nanopore MinION for the detection of antimicrobial resistance genes
Source: J Antimicrob Chemother. 2015 Jul 28;70(10):2775–8. doi: 10.1093/jac/dkv206 (PMC4566964; doi:10.1093/jac/dkv206)
Supplement: Supplementary Data [file supp_dkv206_dkv206supp.docx]

**Supplementary data**

**Supplementary Methods**

*DNA extraction*

Bacterial isolates used in the study were maintained at -80^o^C. Frozen stock was streaked onto Columbia Blood Agar plates, which were incubated at 37^o^C in air overnight. A single colony was picked into 4ml Brain Heart Infusion broth using a sterile 10μL loop, and incubated at 37^o^C in a shaking incubator for 24 hours. DNA extraction was carried out using the QiaAMP DNA Mini kit (Qiagen, Venlo, Limburg, Netherlands) according to the manufacturer’s instructions. DNA was quantified using the Qubit fluorimeter and BR kit (Life Technologies, Paisley, UK) following the manufacturer’s protocol.

*MinION sample preparation*

Genomic DNA was diluted in 10mM Tris-HCl to a concentration of 1000ng in 80μL and sheared using G-TUBES (Covaris, Woburn, MA, USA). Sample preparation was carried out according to the instructions supplied by Oxford Nanopore as part of the MinION Access Program for R7 chemistry, and using the supplied Genomic DNA Sequencing Kit SQK-MAP-002. Control DNA (5μL, Oxford Nanopore) was added to the 80μL sheared DNA then end repair was carried out in a total volume of 100uL by adding 10μL buffer and 5μL enzyme, using the NEBNext End-Repair module (NEB, Hitchin, UK) but reducing the incubation time from 30 to 12 minutes. Clean up was performed on the resulting DNA using 1x (by volume) AMPure XP Beads (Beckman Coulter, High Wycombe, UK) and 80% ethanol. DNA was eluted into 25.2μL nuclease free water and A-tailing carried using 3μL buffer and 1.8μL enzyme from the NEBNext dA-tailing module (NEB) and a reduced incubation time from 30 to 15 minutes. 50μL of Blunt/TA Ligase Master mix (NEB) Adapter Mix (10μL, Oxford Nanopore) and HP adapter (10μL, Oxford Nanopore) were added to the A-tailed DNA and incubated at room temperature for 10 minutes. The sample was cleaned up using 0.4X AMPure XP Beads (Beckman Coulter) using Oxford Nanopore’s recommended modifications to the standard process and eluted in 25μL of elution buffer (Oxford Nanopore). Eluted DNA was mixed with 10μL of Tether reagent (Oxford Nanopore) and incubated for 10 minutes, then mixed with 15μL of HP motor (hairpin motor, Oxford Nanopore) and incubated overnight at room temperature. After incubation this is referred to as the pre-sequencing mix.

*MinION sequencing*

R7 Flow cells were quality controlled using the Platform_QC script and primed twice with 150μL EP buffer (Oxford Nanopore), allowing ten minutes at room temperature after each priming step. 6uL pre-sequencing mix, 4μL Fuel Mix (Oxford Nanopore) and 140μL of EP buffer were mixed, added to the flow cell, and the 48-hour genomic DNA sequencing script run in MinKNOW V0.45.3.9. Metrichor V0.17 was used for basecalling using the R7 1.2 and 1.3 2-directional workflows. MinION sample preparation and loading of the flow cell took approximately two hours and thirty minutes.

*Illumina and PacBio sequencing*

Library construction for Illumina sequencing was carried out as described previously,^1^ using the Kapa Hifi polymerase (Kapa Biosystems, Woburn, MA, USA) for PCR amplification. Libraries for all six isolates were pooled into a single, multiplexed library, which was sequenced on an Illumina MiSeq (Illumina, San Diego, CA, USA) for 151 cycles from each end plus an 8-base index sequence read. PacBio SMRT (single-molecule real-time) sequencing was used to generate long reads of MRSA SASCBU26. A single library was constructed from 2µg of genomic DNA that had been sheared to 10kb with a G-TUBE. Library preparation (SMRTbell) was carried out following the manufacturer's protocol (Pacific Biosciences, Menlo Park, CA, USA). The SMRTbell library was bound with version P4 polymerase, and the subsequent complexes loaded on to V3 SMRTcells using MagBeads. The complexes were immediately sequenced using version C2 chemistry.

*Analysis*

Basecalled MinION reads were converted from FAST5 to FASTQ and FASTA formats using an in-house script. Read mapping of the MRSA isolate was carried out using the BWA-MEM algorithm of BWA v0.7.10.^2^ For short read Illumina data the default mapping parameters were applied. For MinION and PacBio data the PacBio mapping option was used, with a minimum seed length variation of between 7 and 21 to optimise the mapping. The optimal seed length was found to be 17 and 11 for PacBio and MinION data, respectively. For comparison, mapping was also performed using LAST,^3^ using the same parameters (match = +2, mismatch = -5, gap open = -2, gap extend = -1). The lastal alignment produced lower indel rates at the expense of a much higher mismatch rate. Calculation of scaled alignment scores for the alignments produced by BWA-MEM and lastal with an in-house script showed the alignment of BWA-MEM to be superior under the parameters used (mean mapping score per base = 0.279 for BWA-MEM and -0.174 for lastal using the parameters described above), at the cost of a slightly lower number of reads being aligned (15,118 for BWA-MEM vs 20,902 for lastal). We report the results from BWA-MEM alignment due to its higher alignment score and to allow more informative comparisons with the alignments for the other technologies.

Output SAM files from BWA-MEM were converted to sorted BAM files using SAMtools v0.1.19-44428cd,^4^ and mapping statistics calculated using an in-house Python script utilising the pysam library v0.7.5. The presence of resistance genes were identified in Gram-negative bacilli and MRSA using a clustered version of the ARG-ANNOT resistance gene database,^5^ which is available as part of the SRST2 programme.^6^ This was searched against the MinION reads using glsearch v36.3.5e from the FASTA package^7^ to identify matches between the entire length of each gene in the database and local regions of the MinION reads. Matches were filtered to remove those below an e-value cut-off of 1e-100. For each gene, the top matches for each read were aligned using MUSCLE v3.8.31^8^ with the gapopen penalty reduced to -6 to take into account the high indel error rate. A consensus sequence was created from this alignment, where each consensus position was called as the majority base when present in at least 50% of reads. Bases were called as N if at least 50% of reads had a base call at the position, but no individual base reached the 50% cut-off. When 50% or more reads included a gap at a position, no base call was made. The consensus sequence was then aligned to the matched gene using MUSCLE with default parameters, and the %ID calculated as the percentage of matches in the alignment excluding sites with gaps in one of the sequences. For MRSA SASCBU26 the same method was used to identify gene matches in PacBio reads. Illumina data for each isolate was assembled using Velvet v 1.2.09^9^ using the VelvetOptimiser wrapper v2.2.4 with kmers ranging from 99 to 135 and parameters optimised based on N50. Gene matches were identified in the assembly using glsearch with an e-value cut-off of 1e-100 and a %ID cut-off of 70. This stricter cut-off was used to reflect the increased confidence in base calls in the assembly and to reduce spurious low identity matches. Identity values were calculated as for the MinION and PacBio analyses.

All sequence data has been submitted to the European Nucleotide Archive (ENA) (<http://www.ebi.ac.uk/ena/>). The assembly of the PacBio data has been submitted under the BioProject ID number PRJEB7753. MinION and Illumina read data has been submitted under BioProject ID number PRJEB7314. Illumina read data is submitted under accession numbers ERR698913 (AB223), ERR698914 (EC1a), ERR698915 (EC302), ERR698917 (Eco216), ERR698918 (KP652) and ERR698916 (SASCBU26). MinION read data is submitted under accession numbers ERR701171 (AB223), ERR701173 (EC1a), ERR701172 (EC302), ERR701174 (Eco216), ERR701175 (KP652) and ERR701176 (SASCBU26).

1. Quail MA, Smith M, Coupland P *et al.* A tale of three next generation sequencing platforms: comparison of Ion Torrent, Pacific Biosciences and Illumina MiSeq sequencers. *BMC genomics* 2012; **13:** 341.
2. Li H, Durbin R. Fast and accurate short read alignment with Burrows-Wheeler transform. *Bioinformatics* 2009; **25:** 1754-1760.
3. Kiełbasa SM, Wan R, Sato K *et al.* Adaptive seeds tame genomic sequence comparison. *Genome Res* 2011; **21:** 487-493.
4. Li H, Handsaker B, Wysoker A *et al.* The Sequence Alignment/Map format and SAMtools. *Bioinformatics* 2009; **25:** 2078-2079.
5. Méditerranée Infection. Antibiotic Resistance gene-ANNOTation (ARG-ANNOT) <http://en.mediterranee-infection.com/article.php?laref=283&titre=arg-annot-> Last accessed 27 Feb 2015.
6. Inouye M, Dashnow H, Raven LA *et al.* SRST2: Rapid genomic surveillance for public health and hospital microbiology labs. *Genome Med* 2014; **6:** 90.
7. Pearson WR, Lipman DJ. Improved tools for biological sequence comparison. *Proc Natl Acad Sci U S A* 1988; **85:** 2444-2448.
8. Edgar RC. MUSCLE: multiple sequence alignment with high accuracy and high throughput. *Nucleic Acids Res* 2004; **32:** 1792-1797.
9. Zerbino DR, Birney E. Velvet: algorithms for de novo short read assembly using de Bruijn graphs. *Genome Res* 2008; **18:** 821-829.

**Table S1.** Summary of gene cluster matches for each isolate. For each isolate the %ID of matches found in the Illumina assemblies and MinION data are shown. For MRSA SASCBU26 the same statistic is also reported for PacBio data. Carbapenemases, ESBLs and *mecA* are highlighted in green.

See the Excel file for Table S1.

**Figure S1.** Scatter plot of percentage ID of gene cluster matches vs. number of reads used to generate consensus sequence of gene.
